# Hospital-wide access to genomic data advanced pediatric rare disease research and clinical outcomes

**DOI:** 10.1038/s41525-024-00441-9

**Published:** 2024-12-02

**Authors:** Courtney E. French, Nancy C. Andrews, Alan H. Beggs, Philip M. Boone, Catherine A. Brownstein, Maya Chopra, Janet Chou, Wendy K. Chung, Alissa M. D’Gama, Ryan N. Doan, Darius Ebrahimi-Fakhari, Richard D. Goldstein, Mira Irons, Christina Jacobsen, Margaret Kenna, Ted Lee, Jill A. Madden, Amar J. Majmundar, Nina Mann, Sarah U. Morton, Annapurna Poduri, Adrienne G. Randolph, Amy E. Roberts, Stephanie Roberts, Matthew G. Sampson, Diane D. Shao, Wanqing Shao, Aditi Sharma, Eliot Shearer, Akiko Shimamura, Scott B. Snapper, Siddharth Srivastava, Jay R. Thiagarajah, Mary C. Whitman, Monica H. Wojcik, Shira Rockowitz, Piotr Sliz

**Affiliations:** 1 Children’s Rare Disease Collaborative, Boston Children’s Hospital, Boston, MA USA; 2 Division of Genetics and Genomics, Boston Children’s Hospital, Boston, MA USA; 3 Department of Pediatrics, Harvard Medical School, Boston, MA USA; 4 The Manton Center for Orphan Disease Research, Boston Children’s Hospital, Boston, MA USA; 5 Broad Institute of MIT and Harvard, Cambridge, MA USA; 6 Rosamund Stone Zander Translational Neuroscience Center, Boston Children’s Hospital, Boston, MA USA; 7 Division of Immunology, Boston Children’s Hospital, Boston, MA USA; 8 Division of Newborn Medicine, Boston Children’s Hospital, Boston, MA USA; 9 Department of Neurology, Boston Children’s Hospital, Boston, MA USA; 10 F.M. Kirby Neurobiology Center, Boston Children’s Hospital, Boston, MA USA; 11 Division of General Pediatrics, Boston Children’s Hospital, Boston, MA USA; 12 Division of Endocrinology, Boston Children’s Hospital, Boston, MA USA; 13 Department of Otolaryngology and Communication Enhancement, Boston Children’s Hospital, Boston, MA USA; 14 Department of Otolaryngology Head and Neck Surgery, Harvard Medical School, Boston, MA USA; 15 Department of Urology, Boston Children’s Hospital, Boston, MA USA; 16 Department of Surgery, Harvard Medical School, Boston, MA USA; 17 Division of Nephrology, Boston Children’s Hospital, Boston, MA USA; 18 Department of Neurology, Harvard Medical School, Boston, MA USA; 19 Department of Anesthesiology, Critical Care and Pain Medicine, Boston Children’s Hospital, Boston, MA USA; 20 Department of Anaesthesia, Harvard Medical School, Boston, MA USA; 21 Department of Cardiology, Boston Children’s Hospital, Boston, MA USA; 22 Division of Nephrology, Brigham and Women’s Hospital, Boston, MA USA; 23 Department of Hematology and Oncology, Boston Children’s Hospital, Boston, MA USA; 24 Dana Farber Cancer Institute, Boston, MA USA; 25 Division of Gastroenterology, Hepatology and Nutrition, Boston Children’s Hospital, Boston, MA USA; 26 Department of Ophthalmology, Boston Children’s Hospital, Boston, MA USA; 27 Department of Ophthalmology, Harvard Medical School, Boston, MA USA; 28 Division of Molecular Medicine, Boston Children’s Hospital, Boston, MA USA; 29 Department of Biological Chemistry and Molecular Pharmacology, Harvard Medical School, Boston, MA USA

**Keywords:** Paediatric research, Genetics research

## Abstract

Boston Children’s Hospital has established a genomic sequencing and analysis research initiative to improve clinical care for pediatric rare disease patients. Through the Children’s Rare Disease Collaborative (CRDC), the hospital offers CLIA-grade exome and genome sequencing, along with other sequencing types, to patients enrolled in specialized rare disease research studies. The data, consented for broad research use, are harmonized and analyzed with CRDC-supported variant interpretation tools. Since its launch, 66 investigators representing 26 divisions and 45 phenotype-based cohorts have joined the CRDC. These studies enrolled 4653 families, with 35% of analyzed cases having a finding either confirmed or under further investigation. This accessible and harmonized genomics platform also supports additional institutional data collections, research and clinical, and now encompasses 13,800+ patients and their families. This has fostered new research projects and collaborations, increased genetic diagnoses and accelerated innovative research via integration of genomics research with clinical care.

## Introduction

The availability and efficiency of genomic sequencing in the diagnosis of rare monogenic diseases has led to frequent use of genomics in research and gradual adoption in clinical practice. Over the past decade, numerous large rare disease sequencing research studies have characterized the burden of Mendelian disease across various phenotypes^1–4^. The clinical impact of timely genomic sequencing for pediatric patients has been well-established in the critical care setting^5–8^. However, another advancement has been the development of genomics-driven platforms that facilitate research-informed healthcare for broader groups of patients^9–14^ in a variety of care contexts. Critically, research-clinical integration for genomics includes building infrastructure to consent for research sequencing, confirm selected research findings and deliver clinically validated results directly to patients, as well as a mechanism to conduct deeper ongoing analysis in a research setting on non-diagnostic clinical cases. Establishment of a research-clinical cycle leverages the power of research to improve clinical care by filling gaps in diagnostics and accessibility while maintaining the highest standards for clinical diagnosis.

Exome sequencing (ES) has had broad use in rare disease research studies and is increasingly utilized in clinical care. However, genome sequencing (GS) has become more common as the cost of sequencing has decreased. GS has been shown to increase diagnostic yields up to 10% through improved variant calling for small variants, copy number variants (CNVs), and other structural variants (SVs) and the ability to interrogate the non-coding space^15–18^ including in regions of known disease-associated genes. In addition, other genomic technologies, such as long-read GS and transcriptome sequencing, have been deployed to resolve cases undiagnosed by ES/GS^19–22^. However, these other technologies are still mostly limited to research.

The Children’s Rare Disease Collaborative (CRDC) at Boston Children’s Hospital (BCH) was launched in 2018 with the goal of integrating research and clinical genomic data into an accessible genomics platform to drive pediatric precision medicine. Phase I, completed in 2019, included the establishment of an infrastructure for consenting patients and their parents, data sharing, and analysis of ES for 1046 affected individuals across 15 disease cohorts^11^. Here, we describe the results from Phase II and the first five years overall of the CRDC. Having established a large cohort of patients with pediatric rare disease presentations ascertained by subspecialty experts, with deep disease-specific phenotype information and genomic data, we demonstrate the potential of an institutional research-clinical partnership in facilitating new discoveries and advancing pediatric healthcare.

## Results

### Establishing a genomic sequencing ecosystem

**Fig. 1:**
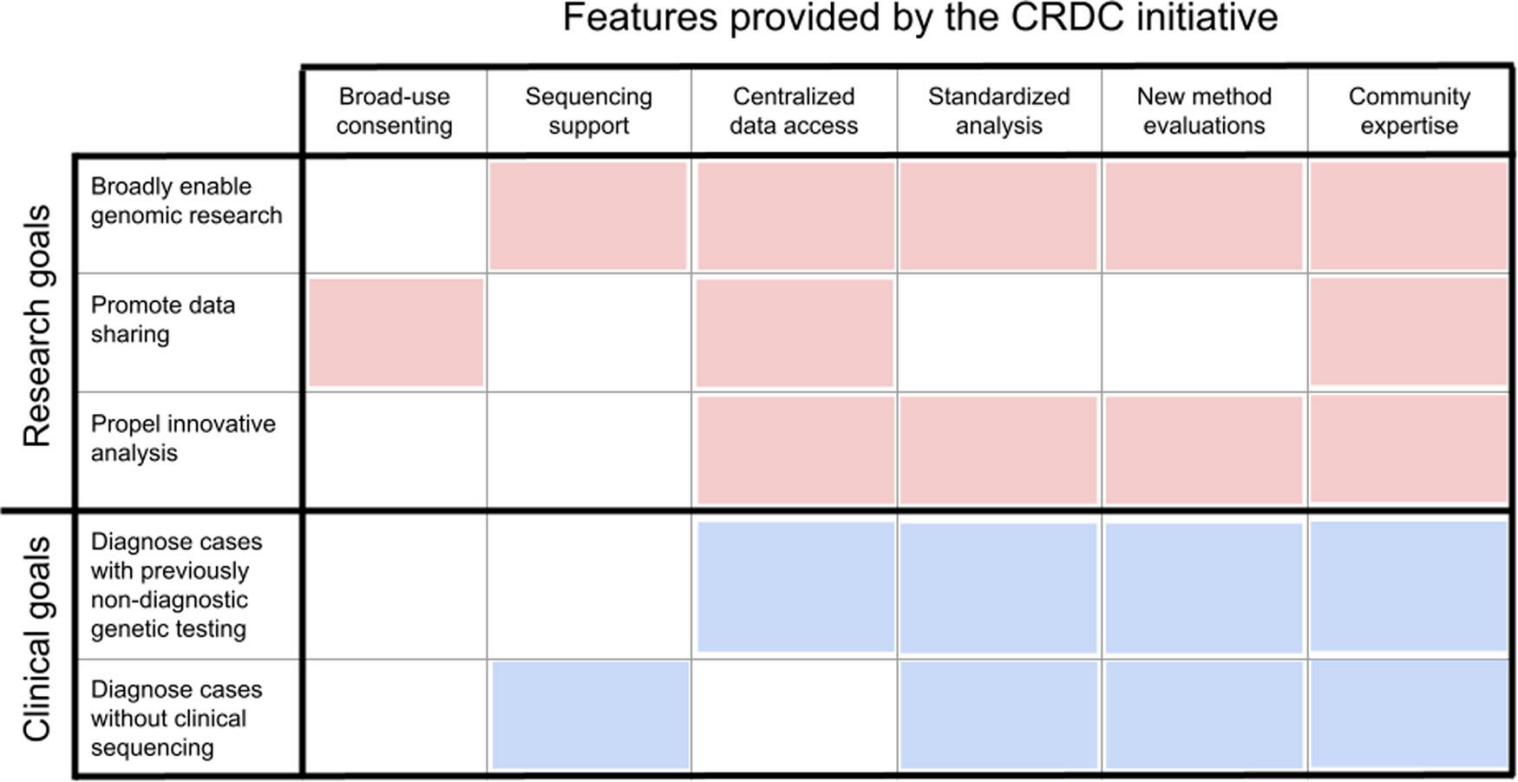
Key features of the CRDC. This chart displays the six key features of the CRDC across the top and how they contribute to its research (red) and clinical (blue) goals. This figure was created in Microsoft PowerPoint.

As previously described, the CRDC was created in alignment with our institutional goals as part of the BCH Research Strategic plan and as the outcome of a Blue Ribbon committee commissioned in 2018^11^. The goal was to establish a scalable, clinical-grade genomic sequencing platform that advances rare disease research and improves clinical care. To this end, the collaborative developed a variety of features and resources for research and clinical communities across the institution (Fig. 1). These features include integrating language for broad-use research and data sharing into consents, extensive financial support for research ES and GS, centralized data access to both research- and clinically-generated sequencing data, a comprehensive and standardized analysis platform, a mechanism to evaluate novel methods and analytics, and a network of investigators with diverse disease-specific expertise. The establishment of these resources has enabled investigators at all career levels to perform genomic research, promoted data sharing internally and externally, propelled implementation of innovative analysis and provided access to new pathways of diagnosis for individuals not able to obtain clinical testing or that had received nondiagnostic results.

The CRDC began offering research genomic sequencing for selected rare disease cohorts in late 2018; in the first year (Phase I), the collaborative generated ES data for 1046 affected individuals across 15 cohorts, developed consent language for broad-use research and implemented a harmonized data processing and standardized analysis platform^11^. Since then, additional disease cohorts were selected for funding about once a year via a hospital-wide call for applications. Cohorts were chosen based on potential for novel discoveries and scientific innovation. Moreover, the selection criteria were inclusive, with a goal to broaden the availability of genomic sequencing across all the divisions and departments and investigators at all career levels. As of early 2024, the CRDC included 45 disease cohorts led by 66 investigators from 26 departments/programs (Table 1). These disease cohorts each covered at least 5 and up to hundreds of different genetic diseases, defined by the Genetic and Rare Diseases Information Center (GARD, rarediseases.info.nih.gov).

**Table 1:** Overview of the disease cohorts involved in the CRDC

Department/Division	Disease cohort	Families with genomic data and consent for broad-use research	Median age of patient at enrollment (years)^a^	Percent that includes both parents (trio sequencing)^a^	Percent of probands with GS^a^	Average number of HPO terms^a,b^	Overall number of families with research genomic data
Neurology	Unexplained Epilepsies	941	9.4	66%	36%	51	1166
	Cerebral Palsy and Related Disorders	265	8.7	55%	13%	75	265
	Hereditary Spastic Paraplegia and Movement Disorders	68	10.0	71%	93%	84	68
	Brain Malformations	43	4.6	77%	33%	68	469
	Cerebrovascular Disorders	21	11.4	52%	0%	47	21
	Agenesis of the Corpus Callosum	0	-	-	-	-	24
Genetics and Genomics	Ultra-Rare Disease	425	7.4	69%	45%	73	1235
	ADHD and Related Disorders	339	10.3	72%	7%	46	339
	Myopathies and Dystrophies	57	10.6	83%	22%	64	456
	Sudden Unexpected Death in Childhood (SUDP/SIDS)	26	0.6	50%	65%	-	486
	Cornelia de Lange Syndrome and Related Disorders	7	10.5	43%	0%	103	7
	Interstitial Cystitis	0	-	-	-	-	354
Endocrinology	Idiopathic Short Stature	91	9.3	56%	2%	56	91
	Connective Tissue Disorders	45	15.6	31%	20%	85	45
	Osteogenesis Imperfecta	38	13.8	61%	0%	51	38
	Disorders of Sex Development and Hypospadias	33	3.7	48%	3%	43	169
	Precocious Puberty	28	9.5	25%	0%	43	28
Cancer and Blood Disorders	Anemias and Iron Disorders	26	8.4	58%	5%	29	332
	Bone Marrow Failure and Leukemia Predisposition	20	3.7	65%	90%	70	282
	Schwamman Diamond Syndrome	3	-	33%	100%	91	3
	Sickle Cell Disease	0	-	-	-	-	974
Gastrointestinal	Inflammatory Bowel Disease	793	15.4	24%	37%	41	811
	Congenital Diarrheas and Enteropathies	94	6.6	22%	30%	57	106
	Intestinal Failure due to Malrotation and Volvulus	13	6.3	62%	100%	40	13
Otorhinolaryngology	Hearing Loss	451	7.2	53%	13%	43	451
	Hearing Loss and Cochlear Implants	62	2.4	3%	45%	41	62
	Peripheral Vestibular Disorders	59	13.4	44%	2%	62	59
Immunology	Immunodeficiencies, Autoimmunity and Immune Dysregulation	291	10.8	44%	8%	67	372
	Severe Pediatric COVID-19 and MIS-C	132	8.3	1%	80%	55	150
	Graves disease	31	17.5	0%	29%	64	31
Pulmonology	Interstitial Lung Disease	220	10.7	35%	26%	65	220
	Bronchiectasis	148	20.7	11%	37%	64	148
Urology	Bladder Exstrophy-Epispadias Complex	87	9.1	41%	21%	45	104
	Disorders of Voiding	12	16.5	25%	0%	55	12
Nephrology	Nephrotic Syndrome and Glomerular Disease	45	9.7	9%	56%	49	251
	Urinary Tract Stone Disease	14	5.4	86%	29%	58	14
Psychiatry	Early-Onset Major Depression	30	13.1	30%	33%	53	30
	Early-Onset Psychosis	7	14.6	14%	0%	51	7
Ophthalmology	Infantile Esotropia	24	5.0	71%	21%	36	24
	Infantile Nystagmus	6	6.3	67%	17%	-	6
Newborn Medicine	Neonatal Critical Illness	16	0.2	31%	63%	67	16
	Complex Fetal Cases	11	fetal	91%	27%	37	11
Intersectional	Congenital Heart Disease and Autism Spectrum Disorder	24	11.1	75%	67%	81	24
Anesthesiology	Severe Chronic Pain and Insensitivity to Pain	22	15.1	68%	9%	52	22
Plastic and Oral Surgery	Ectodermal Dysplasia and Cleft Lip or Palate	1	-	0%	0%	119	1

*HPO* human phenotype ontology, *SUDP* sudden unexpected death in pediatrics, *SIDS* sudden infant death syndrome, *ADHD* attention deficit/hyperactivity disorder, *MIS-C* multisystem inflammatory syndrome in children.

^a^Data for families sequenced through the CRDC with broad-use research consent.

^b^Included HPO terms collected by researchers and extracted from the electronic health record with Clinithink.

Since the launch of the collaborative, the process to onboard new cohorts has been streamlined, particularly at the stage of Institutional Review Board (IRB) review, which was 16% faster for the 15 most recent consents that include standardized CRDC-specific language than the first 12. There have been many improvements in the process of enrolling individuals and collecting samples. Efforts to develop methods of remote consenting and sample collection accelerated during the COVID-19 pandemic to mitigate pandemic-related restrictions on general on-site interactions with patients and research participants. The ability to consent and enroll remotely/electronically and to remotely collect buccal samples (ES only) continues even as clinics and research are permitted to occur on-site.

**Fig. 2:**
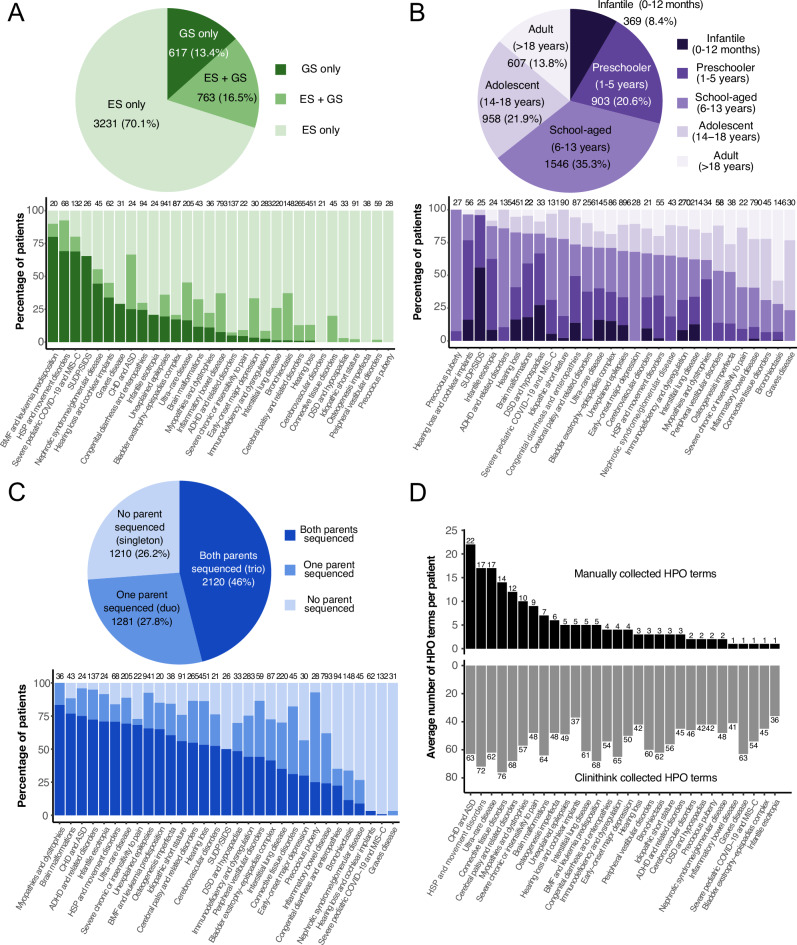
Overview of participants and data included in the CRDC. Overview of participants and data included in the CRDC. **A** Distribution of type of sequencing performed. **B** Distribution of age at enrollment. **C** Distribution of sequencing of parents. For (**A**–**C**), the pie chart includes patients for all CRDC-sequenced cohorts combined and the bar chart includes individual CRDC-sequenced cohorts with at least 20 patients. **D** Average number of HPO (Human Phenotype Ontology) terms collected per patient for individual CRDC-sequenced cohorts with at least 20 patients. Top: HPO terms collected manually by research teams. Bottom: HPO terms extracted from the electronic health record by Clinithink. SUDP sudden unexpected death in pediatrics, SIDS sudden infant death syndrome, ADHD attention deficit/hyperactivity disorder, DSD disorders of sex development, MIS-C multisystem inflammatory syndrome in children, HSP hereditary spastic paraplegia, ASD autism spectrum disorder, CHD congenital heart defect. This figure was created in R with ggplot^39^.

Additionally, in Phase II, the CRDC began supporting GS, in addition to ES, as a sequencing test. The original experimental design was to first perform ES on the proband and available family members and then reflex selected non-diagnostic cases to GS of the proband. However, in March 2022, GS began to be offered as a first-line test to all disease cohorts. Since then, usage of GS has grown, currently accounting for 35% of tests ordered. The majority of tests ordered continues to be ES, however, largely because buccal swabs and thus remote sample collection have only recently been accepted by GeneDx for GS. Overall, 70% of probands have only ES data while 17% of probands have ES + GS data and 13% have GS as the primary test (Table 1, Fig. 2A).

**Fig. 3:**
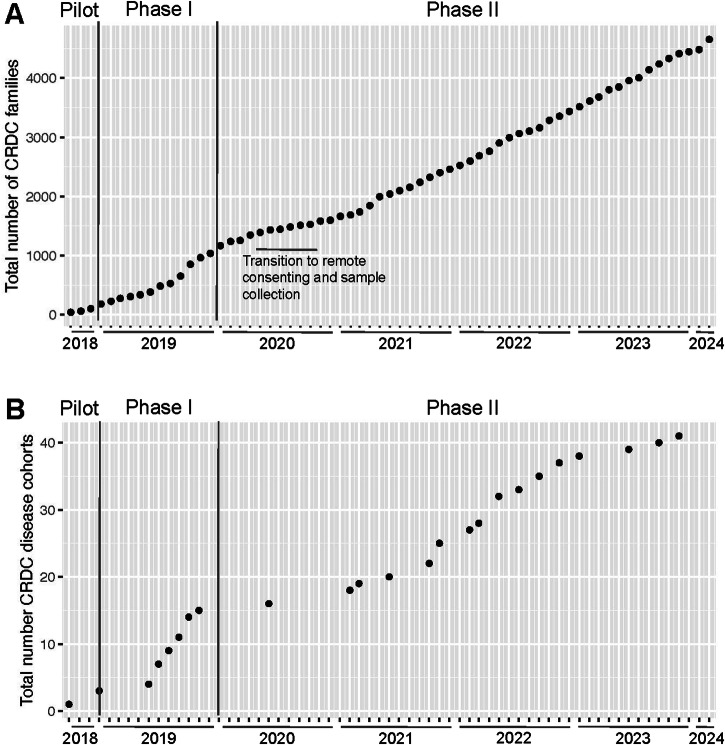
Growth of the CRDC since launch. These plots track the increase in total number of (**A**) families receiving genomic sequencing through the collaborative under a broad-use research consent and (**B**) disease cohorts enrolling such families. Enrollment slowed slightly in the early months of the COVID-19 pandemic as researchers transitioned to remote consenting and sample collection, as marked on the plot. This figure was created in R with ggplot^39^.

As of February 2024, 6308 rare disease patients and their families (13,723 individuals) that consented to a research study have had ES (100x average coverage) and/or GS (40× average coverage) performed via the CRDC, with 4653 of those families (11,150 individuals across 41 disease cohorts) consented for broad-use research purposes and data sharing (Fig. 3). These data have been harmonized in an institution-wide genomics repository with genomic and phenotypic data collected from other research projects and support sources. Additionally, a workflow was established whereby data generated from clinically-ordered sequencing was returned and harmonized in the repository, facilitating clinically-driven re-analysis and reflex to a research study. The repository thus contains 5694 families under a broad-use research consent, 4916 families under other research consents, and 3266 clinically-sequenced families not currently involved in a research study for a total of 31,168 individuals. All data were made available to the appropriate researchers or clinicians in a standardized genomics analysis platform with multiple tools for investigation including GeneDx’s Discovery Platform, Illumina’s Emedgene, a local instance of the Broad Institute’s Seqr platform^23^ and a gnomAD-like browser developed in-house, BCH Aggregator.

The set of CRDC-sequenced data consented for broad research (4,653 families) is described in this report and comprises data from probands who are mostly pediatric (86% with age ≤18 years at time of enrollment, median age = 11 years) (Table 1, Fig. 2B) and are 53% male and 47% female. Where possible, the biological parents of probands and other relevant family members were also consented to the study. Forty-six percent of families (2120) included both biological parents (trios) and another 28% included one biological parent (duos) (Fig. 2C). Ten percent of families included at least one other non-parental family member, 91% of whom included siblings. Seventy-two percent of CRDC probands were self–reported as White, non-Hispanic/non-Latine (compared to 67% of the overall hospital patient population)^24^.

Phenotypic information is collected in a centralized repository for studies involved in the CRDC via two methods. The first is manual entry of clinical information into disease-specific REDCap^25,26^ databases by individual research teams. Each disease cohort had an average of 1–16 Human Phenotype Ontology (HPO) terms per patient (Fig. 2D) and with an overall average across all cohorts of 5 HPO terms/patient. Many research groups also collected additional phenotype information (e.g., EEGs and MRIs) and the disease-specific REDCap databases could have hundreds of fields. The second method of phenotypic data collection is pulling from the electronic health record (EHR) via Clinithink (www.clinithink.com), a natural language processing algorithm. This method resulted in an average of 52 HPO terms per proband (Fig. 2D). All these data were then collected in a single central REDCap repository allowing for easy dissemination to various analysis tools.

### Advancing rare disease research

**Fig. 4:**
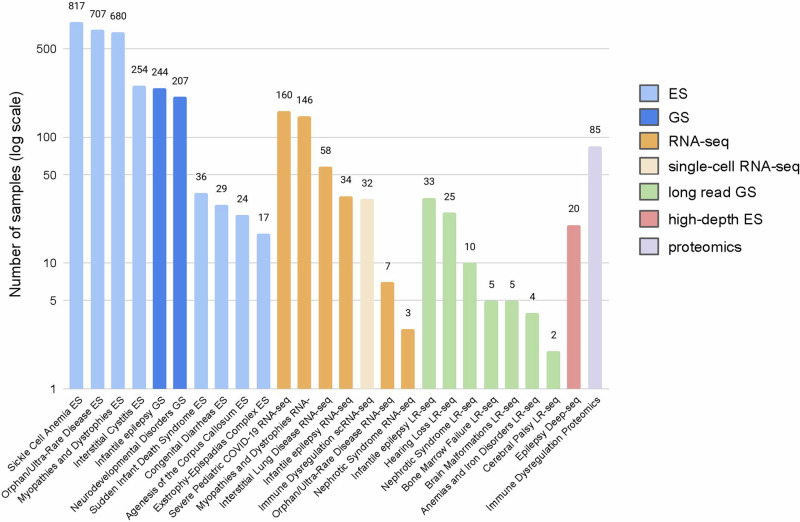
Additional projects supported by the CRDC. A number of samples were included in additional projects performed to increase access to sequencing (ES, GS) and evaluate orthogonal methods for identifying the genetic basis of a rare disease presentation: RNA-seq, single-cell RNA-seq (scRNA-seq), long-read genome sequencing (LR-seq), high-depth exome sequencing to detect somatic mosaic variants (Deep-seq) and proteomics. This figure was created in Microsoft Excel.

In addition to providing sequencing support for studies that prospectively enrolled patients under a broad-use research consent, the CRDC has also supported several projects with the goal of expanding access to sequencing and other diagnostic methods. One major arm of this was providing support for ES/GS and analysis for patients already enrolled in a different study under a non-broad-use research consent (i.e., allowing only for more limited sharing of data). 1846 patients (2771 individuals) have thus been sequenced across nine disease cohorts including sickle cell anemia, orphan/ultra-rare disease, myopathies/dystrophies, neurodevelopmental disorders, and interstitial cystitis^27^ (Fig. 4), resulting in over 100 additional diagnoses so far.

The other major arm was supporting pilot projects to investigate and implement orthogonal experimental methods for identifying the genetic/genomic basis of a rare disease presentation (Fig. 4). One such project involved performing RNA-seq on over 400 samples across seven different cohorts including myopathies, pulmonary disease, severe COVID-19 and epilepsy. Data analysis is ongoing but already a few solved cases have been supported by functional information gleaned from the transcriptomic data, particularly for the COVID-19 and myopathies cohorts, which performed RNA-seq on related tissues: blood and muscle, respectively. Another method investigated was long-read sequencing with data generated for over 80 patients from seven cohorts including hearing loss, epilepsy, and nephrotic syndrome. Preliminary analysis of the data from hearing loss patients has resulted in three additional pathogenic findings undetectable with short-read genome sequencing. Other ongoing projects include testing the utility of single-cell transcriptomics, high-depth exome sequencing for identifying somatic mutations and proteomics in solving rare diseases.

Another major component of this study was creating opportunities for collaboration both within and outside the institution (Table 2). Critical to this was the incorporation of broad sharing for research use into the consent forms. In addition to the 4653 families sequenced via the CRDC, 1041 additional patients and families received genomic sequencing under a consent that allowed for such broad sharing of data. The genomic and phenotypic data for these individuals are available in a de-identified format for cross-cohort analyses in custom in-house tools including BCH Aggregator and Cohort Family Analysis. BCH Aggregator is an integrated database of sequencing data and clinical phenotypes and has a web portal for visualization and exploration. This aggregate resource allows users to investigate how many individuals have variants at a specific locus of interest, which phenotypes those individuals have, the name of the investigator who originally consented the patients and it provides statistics for phenotype-specific burden tests of variants in genes. The Cohort Family Analysis tool aggregates candidate variants produced by family-based prioritization tools across families and cohorts, provides information on variant inheritance, participant phenotype, consenting investigator, allele frequencies, protein impact of variation, protein annotations and protein structures, variant interpretations, splicing, conservation and known gene-disease relationships, and includes various filtering options. Both of these tools facilitate preparatory-to-research queries and building collaborations.

**Table 2:** Data and analysis platforms available for different types of queries through the CRDC

Type of query	Diagnose patients?	Explore a cohort?	Analyze all data? Create a new cohort?			Build collaborations?
Analysis platforms	Family-based analysis with Gregor (GeneDx DP), Emedgene, CFA, BCH Seqr	Cohort-level analysis with CFA, SKAT (GeneDx DP)	Variant summary information via BCH Aggregator	Gene phenotype associations via BCH Aggregator	Find patients with specific variants/genes with CFA, BCH Aggregator	Share variants and phenotypes internally and externally with CFA, BCH Aggregator, MME (BCH Seqr)
Broadly sharable research data (12,677 individuals)	Broadly available	Broadly available	Broadly available	Broadly available	Broadly available	Broadly available
Restricted sharing research data (8813 individuals)	Available only to contributing researcher	Available only to contributing researcher	Broadly available, aggregated only^a^	Broadly available, aggregated only^a^	Via honest broker^b^	Available to contributing researcher and via honest broker^b^
Non-research clinical data (9686 individuals)	Available only to referring clinician	Not available	Broadly available, aggregated only^a^	Broadly available, aggregated only^a^	Via honest broker^b^	Not available

*DP* discovery platform, *CFA* cohort family analysis, *BCH* Boston Children’s Hospital, *SKAT* sequence kernel association test, *MME* matchmaker exchange.

^a^Only aggregated counts are available broadly; it is not possible to assign a specific variant to a specific individual unlike for broadly sharable data, which does so via de-identified IDs.

^b^The CRDC implementation team can connect the querying researcher with the contributing researcher or referring clinician for follow-up and collaboration.

Many disease group researchers reported multiple and diverse collaborations. Internal collaborations have been integral for patient recruitment as many of the patients fall into multiple disease categories and some of the disease groups have unique combinations of phenotypes resulting in new connections between departments in the institution. 624 out of 9797 patients consented to research (6%) are enrolled in multiple research studies. Data can also be shared externally including through Genomic Information Commons^28^. Additionally, through the CRDC, BCH has also been established as a Matchmaker Exchange (MME)^29^ node with the ability to submit data integrated into one of the commonly used analysis tools, an institution-specific instance of the Broad’s Seqr^23^.

The CRDC has also been a critical component of the success of a recent international collaboration between four leading pediatric hospitals to investigate the diagnostic and clinical utility of rapid trio GS in infantile epilepsy^30^. This study recently published the results of the first 100 infants enrolled (43% diagnostic rate), 34 (34%) of whom were enrolled from BCH and supported by the CRDC. In addition to sequencing support, the BCH arm of the collaboration was able to rely on the established workflows and infrastructure to quickly get the study running. Of the published 34 cases, 44% had diagnostic findings, and this study is ongoing with 91 families enrolled at BCH (Fig. 4). The results from this rapid sequencing study have driven changes in standards of care such as offering genomic testing to patients with infantile epilepsy.

### Improving clinical care

**Fig. 5:**
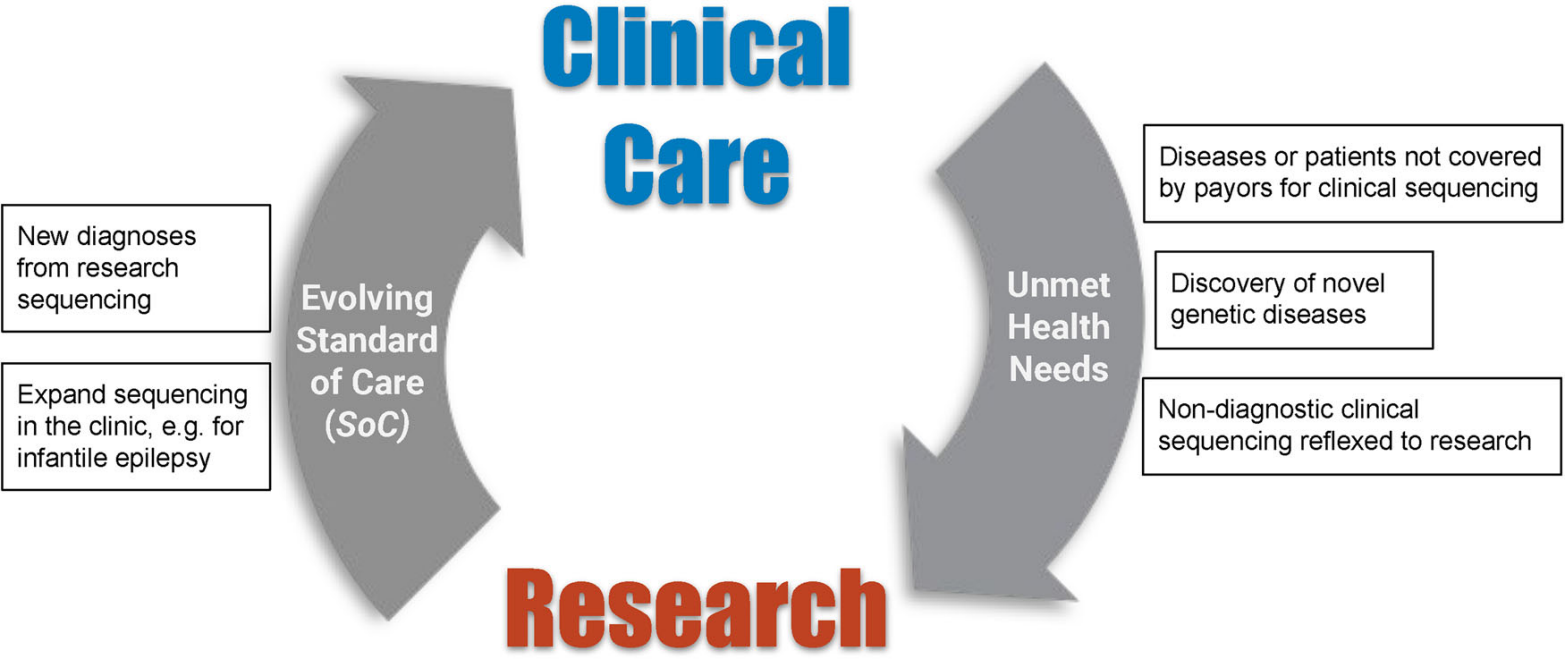
The research-to-clinical loop. The research-to-clinical loop involves taking advantage of the benefits and flexibility of research studies to close the gap on unmet health needs and then evolve the standard of care by bringing the results back to the clinic. This figure was created in Microsoft PowerPoint.

The genomics analysis platform developed by the CRDC drives a research-to-clinical loop where research genomic sequencing can have an immediate impact on clinical care (Fig. 5) because of the built-in framework to clinically confirm findings discovered by researchers and the ability for clinically generated sequencing to be easily re-analyzed or reflexed to research for further investigation. Primary variant analysis for research sequencing was performed by the research group that enrolled the participants and varied depending on the disease, researcher and analysis platform. Generally, filtering was done for population frequency, functional impact and family inheritance where applicable. Some groups used a gene list to help prioritize variants but analysis generally went beyond strict filtering on known disease genes to facilitate novel discoveries. Various annotations and filters are available via the GeneDx Discovery Platform and BCH Seqr, and AI-powered variant prioritization is available via Emedgene. Putative causative variants were then clinically confirmed via validation and classification by the CLIA-certified testing facility GeneDx and returned via the referring clinician.

As of August 2023, 1165 patients enrolled in a CRDC research study (35% of 3353 cases analyzed at that point) had a genetic finding of interest including variants of uncertain significance (VUS) and variants in candidate disease genes, which were slated for follow-up functional analysis, and variants that were clinically confirmed (pathogenic, likely pathogenic or VUS) and returned to the patient’s health record (514 cases, 15% of cases analyzed). Crucially, the ability to clinically confirm research findings is made widely available as the samples are stored in a CLIA environment and the cost of the confirmatory testing by the sequencing lab (GeneDx) is covered by the CRDC.

Alongside research sequencing, clinical sequencing is also available at our center with 4032 patients having been tested from late 2019 through February 2024 (almost all ES with GS only becoming available very recently). The uptake of clinical exomes for patients seen in the clinic increased from 24 per month in 2016–2017 to 90 per month in 2021–2023, mirroring the increase in research genomic sequencing. Since 2019, these sequencing data have also been made available for analysis in the genomics platform, supporting re-analysis by clinicians. Additionally, storing the data in a centralized database makes it available for deeper research investigations if the individuals are enrolled in a research study (1324 of patients with clinical ES are also enrolled in a research study).

To further explore the benefit of accessible research genomic sequencing, a deeper review was performed for four cohorts: epilepsy^31^ (522 patients), hearing loss^32^ (218), cerebral palsy^33^ (175) and peripheral vestibular disorders (32). For all groups, a fraction of enrolled patients had had non-diagnostic previous genetic testing: 40% of the cerebral palsy cohort (mix of CMA and ES), 31% of the hearing loss cohort (mostly panel and single gene tests), 26% for peripheral vestibular disorders (single gene, panel, and ES) and 19% for epilepsy (clinical panel and/or CMA). These cases were included in the studies as the opportunity to perform ES or GS presented an improvement over the previously available tests, often limited to gene panels or CMA.

Another feature of these cohorts was that many of the patients included had phenotypes not classically offered genomic testing such as ES/GS, and for whom insurance coverage for such testing is not routine. In the epilepsy cohort, 73% of patients had an epilepsy diagnosis other than developmental and epileptic encephalopathies (DEE), and had a diagnostic rate of 14% (compared to 32% for DEE). In hearing loss (HL), 45% of patients had unilateral or asymmetric bilateral HL, which historically have not been tested genetically. In this study, 20% of those patients had diagnostic results (compared to 40% of patients with symmetric bilateral HL). Similarly, for cerebral palsy (CP), 48% had non-cryptogenic CP (patients with known acquired risk factors for CP) and a diagnostic rate of 10% compared to the 42% that had cryptogenic CP (with no known risk factors) and a diagnostic rate of 39%. And for the vestibular disorders cohort, where the contribution of Mendelian variants is largely unknown, there have been few variants clinically confirmed and returned so far, but there are a number of ongoing investigations for this gene discovery-focused study.

## Discussion

Genomic sequencing has been established as a critical tool in the diagnosis of pediatric rare diseases. In this study, we described the results of five years of the CRDC, including the enrollment and sequencing of 4653 families under a broad-use research consent and from 41 different rare disease cohorts. 35% of the analyzed cases had findings of interest with 15% clinically confirmed and returned to the family. The rate of clinically confirmed findings is on the low end of published numbers for rare diseases because of ascertainment bias including enrolling patients with non-diagnostic genetic test results and presentations with less well-established genetic etiologies. Even cohorts of the same disease can have different diagnostic rates due to the underlying severity and previous investigations. As a quaternary referring center, we often see particularly complex cases that are being evaluated via several different approaches. Generally groups are not returning secondary findings, although the possibility to do so is included in the standardized consent form. Depending on the disease and research group, variant analysis can be quite broad and secondary findings are not explicitly excluded, allowing for identification if the phenotypes overlap with the primary indication. The logistics of classifying and returning secondary findings from research sequencing continues to be an area under development.

The collaborative has expanded greatly since our first report in 2020^11^, successfully integrating the research and clinical domains, allowing for research genomic findings to have immediate clinical impact. Research groups reported that clinically-confirmed diagnoses resulted in a change in clinical care in up to 25% of cases including changes in treatment, surveillance, change in prognosis and enrollment in a clinical trial. Further, as expected, investigators reported that a precise genetic diagnosis often clarified reproductive risk and allowed families to connect with disease-specific communities for support and information. The availability of research genomics also broadened the cohort of patients who can access genomic testing, including those with diseases for which a Mendelian basis is still under evaluation. Thus, research genomic sequencing improves access to testing for families not historically referred for clinical genomic testing, sharing the benefits of genomic precision medicine with a larger community. While the availability of institution-supported research genomic testing helps address one of the barriers to more equitable access to sequencing across populations, many more obstacles need to be overcome as is described in a recent publication that reviewed racial and ethnic representation in both clinical and research genomic testing performed at BCH^24^.

The CRDC was established as an internally funded program as part of the institution’s research strategic plan and the successes to date indicate a path to sustainability. One example is the cost savings from alignment and standardization of genetics sequencing programs, which greatly reduces data duplication and allows for taking advantage of technologies at scale. Additionally, the data and infrastructure supplied by the CRDC escalates incoming grant funding providing a return on investment. Finally, the CRDC and the work of its associated investigators has made BCH a more specialized and effective rare disease center that, among other benefits, is opening new pathways to innovation in the therapeutic space.

As diagnostic rates increase and sequencing costs fall, hospitals are considering scaling up sequencing efforts and associated cost implications increase from hundreds of samples to thousands or tens of thousands of samples. Choosing between ES and GS then becomes a complex calculation. If we estimate GS costs approximately twice as much as ES, then GS would need to provide twice the number of genetic findings as ES to be financially efficient, which has not yet been demonstrated. One caveat to that statement is that a negative ES test often comes with additional costs as other orthogonal testing may be ordered. Additionally, there are other considerations when choosing ES or GS beyond financial cost and diagnostic rate, and these will vary considerably between patient cohorts. GS can result in increased success particularly in discovering pathogenic variants that are amenable to current precision medicine therapeutics that are being developed, for example, anti-sense oligonucleotides that target deep intronic splice variants only identifiable with GS^34^. In addition, there may be some presentations with a higher probability of being caused by variants that can only be found with GS such as repeat expansion diseases. However, broadly offering GS can limit the access of some patients to the benefits of sequencing as there is generally going to be a cap on the funding available, either from payers and what they are willing to cover or from research and institutional grants. Using the higher-cost option reduces the total number of patients that can receive any form of genomic sequencing, and this is likely to particularly affect those patient groups that are already less likely to have access. Ultimately, establishing a system for disease-specific decision-making processes for choosing ES or GS would help influence policy with insurance/payers.

A major impact of the CRDC has been the acceleration in the use of genomics in different departments of the hospital. This was done by supporting early investigators who may otherwise have had difficulties setting up cohorts and collaborations, which lowers the barrier of access to these research opportunities, improves career development and results in widespread skill improvements of the workforce in genomics. For instance, CRDC lead investigators include, in addition to senior investigators with independent research laboratories, junior to mid-level physician-scientist faculty as well as expert clinicians. For over half of these faculty, participation in the CRDC represents their first major study involving genomics. Our approach to data analysis involved centralized data processing and storage and distributed variant analysis workflows, which provided a higher baseline level of data processing and analysis for research cohorts that may not have their own resources but requires centralized resources to implement. Investigators still have to supply the workforce required for consenting, genetic counseling and result delivery, however, which remains a limitation.

Having a centralized processing platform allowed for switching to an updated reference genome and rolling out new variant calling methods in a systematic and comprehensive manner. The established infrastructure allowed for the rapid initiation of a pediatric severe COVID-19 cohort in the early days of the pandemic, facilitating urgent high-impact research. It has also enabled the swift rollout of a rapid-turnaround hybrid clinical/research trio GS study for infants with epilepsy, part of the International Precision Child Health Partnership (IPCHiP)^30^. The data repository combined with including broad research sharing in the consent forms also facilitated collaboration with ~5% of participants enrolled in multiple CRDC-aligned studies.

The availability of the clinical sequencing data has inspired a push for developing a workflow for systematic re-analysis at the institutional level. This would include routine review of all cases, automated where possible, in order to discover newly reported variants, variants in newly reported genes, and newly relevant variants/genes due to the evolving phenotypes of these pediatric patients. This is facilitated by the fact that the genomic and phenotypic data for five years of patients receiving clinical genomic sequencing can be found in the centralized platforms supported by the CRDC. Also required is a workflow for communicating variants of interest to the referring clinicians and providing access to the data. There is ongoing work to develop these workflows in an accessible and efficient manner, using CRDC-supported platforms such as BCH’s instance of Seqr.

The CRDC is well-poised to continue to adapt new technologies and methods to improve the process of diagnosing and treating pediatric rare diseases. In addition to expanding the experimental methods supported (e.g. long-read sequencing and RNA-seq), the collaborative will continue to work to expand access to these tools by further streamlining enrollment and sample collection. Incorporating new methods such as machine learning algorithms for genomic analysis and patient selection is a potential future path, as is building a bridge from diagnosis to clinical care and novel therapeutics. Investigators continue to leverage the rich phenotypic and genotypic datasets available through this collaborative to improve pediatric outcomes through research-informed healthcare.

## Methods

Boston Children’s Hospital’s CRDC began on October 1, 2018 and is ongoing. The initial study design and many of the core methods have been previously published in a manuscript documenting the Pilot phase and Phase I of the collaborative^11^. The present report includes Phase II, which ran from October 1, 2019 through February 28, 2024. Participant enrollment, sample collection, sequencing, and data analysis were performed as previously described with a few modifications. The Boston Children’s Hospital Institutional Review Board approved all research related to this study, which complied with all relevant ethical regulations including the Declaration of Helsinki, and informed consent was obtained from all research participants and/or their legal guardians. Study data were collected and managed using REDCap electronic data capture tools hosted at BCH^25,26^. In March 2022, the CRDC began supporting GS in addition to ES. Similar to ES, GS was performed by GeneDx (Gaithersburg, MD), a CLIA-certified testing facility; while sequencing was conducted on a research basis, additional DNA was stored for possible clinical confirmation. DNA library preparation was performed with Illumina DNA PCR-Free Prep Tagmentation and followed by 2 × 150 paired-end sequencing on the Illumina NovaSeq 6000 platform to an average depth of coverage of at least 40X.

The centralized genomic data analysis platform was also updated in Phase II. Starting in May 2022, all genomic data were aligned to reference genome build GRCh38 with the DRAGEN (v3.9) secondary analysis platform from Illumina^35^. This included re-aligning all historical data sets. Identification of the following variant types was performed in Phase II: single nucleotide variants and small insertions/deletions (SNV/indels; DRAGEN), CNVs (DRAGEN), SVs (DRAGEN), disease-associated short tandem repeat expansions (STRs; Expansion Hunter^36^ via DRAGEN), mitochondrial genome variants (Mutect2^37^) and mobile element insertions (MEIs; xTEA^38^). Variants were made available to researchers through commercial and in-house analysis platforms.

## Data Availability

The data are available internally to all BCH researchers and clinicians through the genomics analysis platform. To facilitate external access, the CRDC data has also been made available to the Genomic Information Commons project (https://www.genomicinformationcommons.org/). Code available upon request.
